# Predicting Genitourinary Injuries in Polytraumatized Patients—Development of the GUIPP Scoring System

**DOI:** 10.3390/jcm12237341

**Published:** 2023-11-27

**Authors:** Olivia Mair, Michael Müller, Philipp Rittstieg, Philipp Zehnder, Rolf Lefering, Peter Biberthaler, Maren J. Wenk, Marc Hanschen, The TraumaRegister DGU

**Affiliations:** 1 Department of Trauma Surgery, School of Medicine, Klinikum rechts der Isar, Technical University of Munich, 81675 Munich, Germanyphilipp.rittstieg@mri.tum.de (P.R.); peter.biberthaler@mri.tum.de (P.B.); marc.hanschen@mri.tum.de (M.H.); 2 Faculty of Health, IFOM—Institute for Research in Operative Medicine, University Witten/Herdecke, 51109 Cologne, Germany; rolf.lefering@uni-wh.de; 3 Department of Urology and Urological Surgery, University Medical Center Mannheim, 68135 Mannheim, Germany; maren.wenk@umm.de; 4 Committee on Emergency Medicine, Intensive Care and Trauma Management (Sektion NIS) of the German Trauma Society, 80538 Munich, Germany; support-tr@auc-online.de

**Keywords:** genitourinary injuries, urogenital injuries, severe injury, scoring system, predicting injury

## Abstract

Background: The genitourinary system is not as commonly affected as many other organ systems in severely injured patients. Although a delayed and missed diagnosis of genitourinary injuries (GUIs) can severely compromise long-term outcomes, these injuries are frequently overlooked. Therefore, we present a scoring system designed to assist emergency physicians in diagnosing GUIs in severely injured patients. Methods: The data were obtained from the TraumaRegister DGU^®^ from the years 2015–2021. All severely injured patients (ISS ≥ 16) ≥16 years of age and treated in Germany, Austria, or Switzerland were included in this study. We excluded patients who were transferred out early (48 h), and all patients with isolated traumatic brain injury. After the univariate analysis of the relevant predictive factors, we developed a scoring system using a binary logistic regression model. Results: A total of 70,467 patients were included in this study, of which 4760 (6.8%) sustained a GUI. Male patients (OR: 1.31, 95% CI [1.22, 1.41]) injured in motorcycle accidents (OR: 1.70, 95% CI [1.55, 1.87]), who were under 60 years of age (OR: 1.59, 95% CI [1.49, 1.71]) and had sustained injuries in multiple body regions (OR: 6.63, 95% CI [5.88, 7.47]), and suffered severe pelvic girdle injuries (OR: 2.58, 95% CI [2.29, 2.91]) had the highest odds of sustaining a GUI. With these predictive factors combined, a novel scoring system, the GUIPP score, was developed. It showed good validity, with an AUC of 0.722 (95% CI [0.71; 0.73]). Conclusion: Predicting GUI in severely injured patients remains a challenge for treating physicians, but is extremely important to prevent poor outcomes for affected patients. The GUIPP score can be utilized to initiate appropriate diagnostic steps early on in order to reduce the delayed and missed diagnosis of GUI, with scores ≥ 9 points making GUIs very likely.

## 1. Introduction

Trauma-related injuries are the leading cause of death in the younger population ≤ 45 years of age. In addition, traumatic injuries are a major cause of morbidity and a major socioeconomic burden due to the loss of productive working years and high costs of treatment [1]. In recent years, a great deal of research has been conducted to improve the treatment of severely injured patients, thereby improving outcomes and reducing economic costs [2].

According to the literature, genitourinary injuries (GUIs) are present in approximately 10% of adult polytrauma patients [3,4]. The majority of GUIs in industrialized countries are due to blunt trauma such as falls from great heights or road traffic accidents [1]. Men are three times more likely than women to suffer from GUI in polytrauma [2,4].

The kidneys are the most commonly affected urogenital organ injured due to trauma and are very sensitive to deceleration trauma [3]. Although the bladder and urethra are well protected within the pelvic girdle, this close anatomical relation is often responsible for injury to these organs, as shear forces and bone fragments can cause extensive injury such as bladder perforation or urethral disruption. According to the literature, approximately 1–8% of patients with pelvic girdle injuries also sustain lower urogenital tract injuries [2,3,5]. Additionally, the likelihood of GUI increases significantly depending on the severity of the pelvic girdle injury [6].

Although GUIs are rarely the primary cause of death in severely injured patients, they play an important role in the treatment and outcome after polytrauma. GUIs are associated with a prolonged length of hospital stays, increased complication rates, and several long-term morbidities such as renal dysfunction, urethral strictures, or sexual dysfunction [3,7,8,9,10].

GUIs are frequently overlooked during the primary assessment of trauma patients, as more obvious injuries can mask GUI at first [11,12]. Additionally, initial whole-body computed-tomography (WBCT) may be inconclusive regarding GUI, as the delayed urographic phase required to fully assess the urogenital tract is not routinely performed in the emergency setting [1]. Therefore, many authors have attempted to identify predictive factors for the early diagnosis of GUI in trauma patients [5,13]. While experts agree that gross hematuria is a major sign for the presence of GUI, it is not sufficient as a sole predictive factor, as many patients with GUI do not initially present with gross hematuria at first [1,3].

Attempts have been made to predict GUI in polytrauma patients, but these have mostly included indirect signs such as flank hematoma, rapid deceleration trauma, and symphyseal diastasis [3,5,14].

Most recently, the “urotrauma in polytrauma patients with pelvic and/or spinal injuries” (UPPS) scoring system was introduced, which intended to combine the identified risk factors within one scoring system. With an area under the curve (AUC) of 0.843 and a high specificity of 93.4%, the UPPS scoring system was able to achieve a high predictive value for the prediction of GUI [6]. However, the UPPS score came with limitations. It solely focuses on patients with present pelvic and/or spinal injuries; furthermore, the score has not been validated yet.

The purpose of the present study was to give treating physicians in the emergency setting an easy-to-use and robust tool for predicting GUI in severely injured patients at hand. Therefore, modules of the existing UPPS scoring system were validated in a large patient cohort, available through the TraumaRegister DGU^®^ (TR-DGU) of the German Trauma Society, and the score was refined by correcting its flaws.

The application of this novel scoring system is aimed at decreasing the delayed and missed diagnosis of GUI in severely injured patients significantly, enabling the expedition of the correct management of GUI early on.

## 2. Materials and Methods

### 2.1. Data Collection

Data was obtained from the TraumaRegister DGU^®^. The TraumaRegister DGU^®^ of the German Trauma Society (Deutsche Gesellschaft für Unfallchirurgie, DGU) was founded in 1993. The aim of this multi-center database is a pseudonymized and standardized documentation of severely injured patients. Data are collected prospectively in four consecutive time phases from the site of the accident until discharge from hospital: (A) pre-hospital phase, (B) emergency room and initial surgery, (C) intensive care unit, and (D) discharge. The documentation includes detailed information on demographics, injury pattern, comorbidities, pre- and in-hospital management, course on intensive care unit, relevant laboratory findings including data on transfusion, and outcome of each individual. The inclusion criterion is admission to hospital via the emergency room, with subsequent ICU/ICM care or reaching the hospital with vital signs and dying before admission to ICU. The infrastructure for documentation, data management, and data analysis is provided by the AUC—Academy for Trauma Surgery (AUC-Akademie der Unfallchirurgie GmbH, Munich, Germany)—a company affiliated to the German Trauma Society. The scientific leadership is provided by the Committee on Emergency Medicine, Intensive Care and Trauma Management (Sektion NIS) of the German Trauma Society. The participating hospitals submit their data pseudonymized into a central database via a web-based application. Scientific data analysis is approved according to a peer review procedure laid down in the publication guideline of TraumaRegister DGU^®^. The participating hospitals are primarily located in Germany (90%), but a rising number of hospitals in other countries contribute data as well (at the moment, from Austria, Belgium, China, Finland, Luxembourg, Slovenia, Switzerland, The Netherlands, and the United Arab Emirates). Currently, more than 28,000 cases from almost 700 hospitals are entered into the database per year. Participation in TraumaRegister DGU^®^ is voluntary. For hospitals associated with TraumaNetzwerk DGU^®^, however, the entry of at least a basic data set is mandatory for reasons of quality assurance.

The present study is in line with the publication guidelines of the TraumaRegister DGU^®^ and registered as TR-DGU project ID 2020-016. Patient information was double pseudonymized and deidentified prior to analysis. Data in the TraumaRegister DGU^®^ are pseudonymized and routinely collected clinical data obtained from the patients’ chart. Patient consent is obtained prior to entering patients’ data into the TraumaRegister DGU^®^; data collection without patient consent is not possible. This study has also been approved by the ethics committee of the medical faculty of Technical University Munich (TUM), Germany (Project number: 2023-180-S-KK).

### 2.2. Patient Population

**Figure 1:**
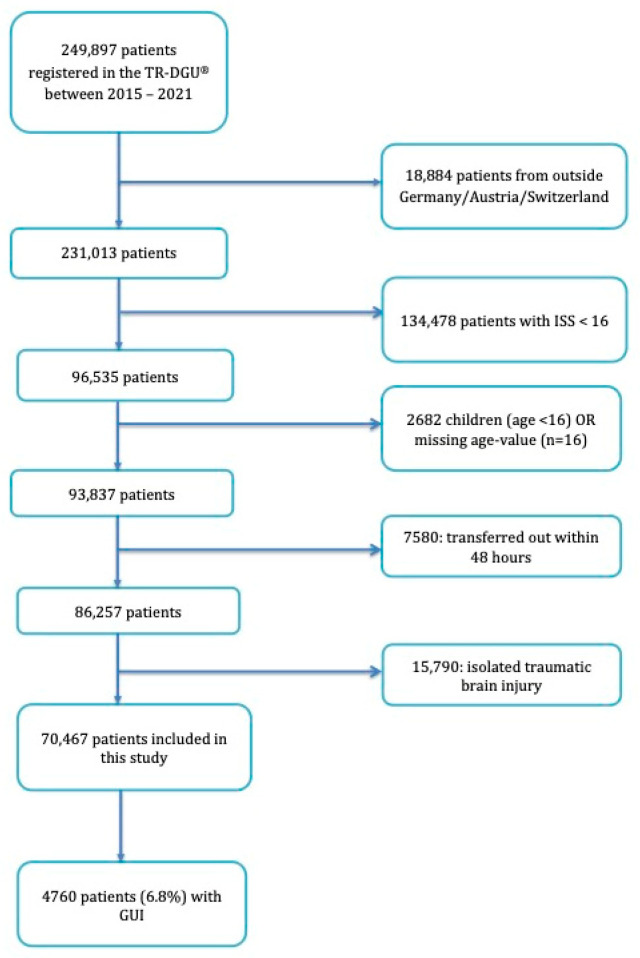
Flowchart of patient enrolment in this study. GUI: genitourinary injury; TR-DGU: TraumaRegister DGU^®^; ISS: Injury Severity Score.

We included all patients registered in the TR-DGU between 2015 and 2021. Patients treated outside Germany, Austria, and Switzerland were excluded to ensure best comparability of treatment. We only included severely injured patients with an Injury Severity Score (ISS) ≥ 16 and excluded children <16 years of age. We also excluded all patients with missing age values in the data set. Additionally, we excluded patients who were transferred out of the initial hospital early on (within the first 48 h) as data entry is expected to be incomplete and documentation of the patient is entered from the receiving hospital. We also excluded all severely injured patients with an isolated traumatic brain injury in order to focus on patients with multiple injuries. Patient enrollment can be seen in Figure 1.

Severity of injuries was classified using the Abbreviated Injury Scale (AIS). It is a grading system, which categorizes injury severity from 1 (=minor injury) to 6 (=maximum/fatal injury). There are no pelvic injuries with an AIS of 1 and moderate pelvic injuries (AIS = 2) comprise stable type A or acetabular fractures. Serious pelvic injuries (AIS = 3) include partially unstable type B pelvic ring fractures, while severe pelvic injuries (AIS = 4) include all unstable type C fractures (e.g., vertical shear and dislocation). Heavy blood loss over 20% of the blood volume or open fractures will increase the AIS category by one level [15]. GUIs were then identified using the ICD-10 codes used to input data in the registry. All injuries to the kidneys (incl. glands), ureters, bladder, urethra, uterus, and vagina and external genitalia were used in this study.

### 2.3. Statistical Analysis

Demographic patient characteristics are presented using mean and standard deviation (SD) for continuous variables, and absolute numbers and percentages for categorial variables. Pearson’s chi-square test or *t*-tests were used to determine differences between patients with and without GUIs. Due to the very large patient cohort, even slight differences would formally become statistically significant. Therefore, only *p*-values that are relevant are given for the interpretation of the results. In general, two-sided *p*-values < 0.05 were considered statistically significant. Parameters which showed a relevant difference in the univariate analysis were then entered into a binary logistic regression model to identify independent risk factors for sustaining a GUI. Results are presented as odds ratios (OR) with 95% confidence intervals (95% CI). Secondly, we awarded points to each category of a significant predictor (except the reference category) to form a simple scoring system. The points awarded were determined by the ORs. The newly developed scoring system was then examined using a receiver operating characteristic curve (ROC) with the area under the curve (AUC) with a 95% CI. We conducted data analysis using the Statistical Package for Social Sciences (SPSS, version 28; IBM Inc., Chicago, IL, USA). For generating graphs, GraphPad Prism Version 9.2.0 (GraphPad Software, LLC. Boston, MA, USA) was used. When presenting numbers to compare the patient groups with and without GUI, the cohort with GUIs will always be named first.

## 3. Results

### 3.1. Patient Characteristics

We were able to include 70,467 patients from 711 hospitals in our study population, of which 6.8% (*n* = 4760) patients sustained GUIs (Figure 1). A total of 3607 (5.1%) patients sustained kidney injuries (incl. glands), 105 (0.1%) patients sustained injuries of the ureters, 608 (0.9%) patients sustained bladder injuries, 263 (0.4%) patients sustained urethral injuries, 47 patients (0.1%) sustained injuries of the uterus or the ovaries, and 354 (0.5%) patients sustained injuries of the outer genitals. Significantly more patients with GUI were male (77.2% vs. 71.3%), *p* < 0.001.

**Figure 2:**
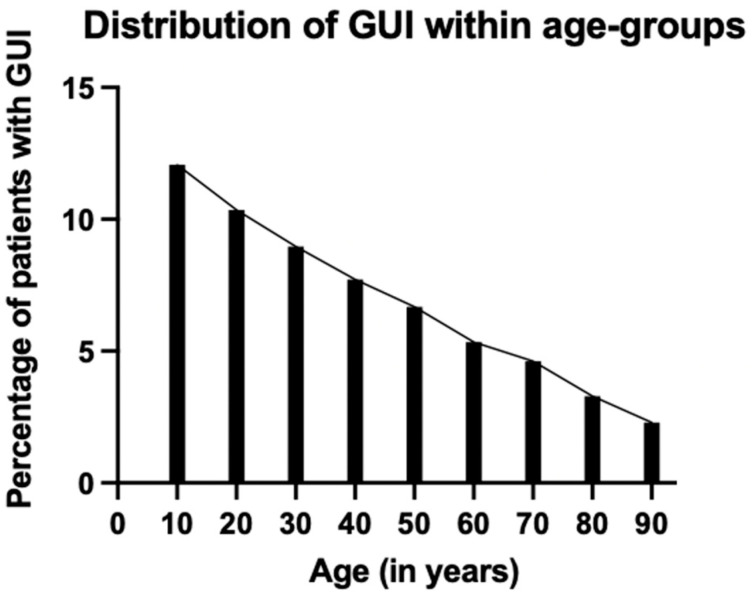
Bar graph showing the almost linear decline of frequency of genitourinary injuries (GUIs) with age.

Overall, the patients with GUIs were considerably younger than patients without GUIs (46.8 ± 20.1 years vs. 55.1 ± 20.8 years, *p* < 0.001). Only 6.4% of the GUI patients were >80 years compared to 14.3% without GUIs. When analyzing age further, there was a highly noticeable, almost linear decline in the relative frequency of GUI with age (Figure 2).

The trauma mechanism presents another important factor in sustaining GUIs. GUIs were mostly present in patients after motorcycle accidents (24.7% vs. 13.0%), followed by car accidents (21.4% vs. 20.9%), and falls from great heights (≥3 m) (19.8% vs. 18.9%). However, only the difference in GUI due to motorcycle accidents is markedly significant when comparing the groups (*p* < 0.001).

Overall, injury severity was significantly higher in patients with GUI (ISS 30.8 ± 13.1 vs. 26.6 ± 10.6, *p* < 0.001). Additionally, we analyzed the number of injured body regions with at least moderate injuries (Abbreviated Injury Scale (AIS) ≥ 2). Therefore, the body was divided into the nine body regions in accordance with the AIS chapters, but the pelvic girdle was separated from the lower extremities in order to have an even more detailed description of the injury pattern. With an increasing number of affected body regions, the prevalence of GUIs increased significantly (*p* < 0.001): at least three body regions (5.3%), at least four body regions (9.1%), at least five body regions (14.4%) and six or more body regions (22.9%).

As expected, GUIs were less likely to be present in patients with leading head injuries (36.1% vs. 53.2%), but more likely in patients with lower extremity injuries (36.1% vs. 28.8%) and pelvic injuries (45.0% vs. 24.2%). No relevant difference was seen with thoracic, spinal, and upper extremity injuries.

Additionally, the higher the severity of the pelvic injury measured by AIS was, the higher was the prevalence of GUIs: 8.4% of patients with moderate pelvic injuries (AIS = 2), 11.0% with serious pelvic injuries (AIS = 3), 13.1% of patients with severe pelvic injuries (AIS = 4), and 18.7% with critical pelvic girdle injuries (AIS = 5).

In order to fully appreciate the relevance of GUIs for severely injured patients, we also analyzed the course of hospital treatment and outcome, although they did not have any direct influence on the development of our scoring system.

The patients with GUIs were treated significantly more often (94.3% vs. 91.0%) and longer (11.3 ± 15.3 days vs. 8.4 ± 12.0 days, *p* < 0.001) in the intensive care unit (ICU). Also, the overall length of hospital stay was longer for patients with GUIs (23.9 ± 23.0 days vs. 18.8 ± 19.4 days). Significantly more patients developed sepsis (11.1% vs. 7.8%) and multi organ failure (MOF) (32.3% vs. 26.1%, *p* < 0.001).

Patients with GUIs were significantly more likely to be treated in level 1 trauma centers (73.6% vs. 68.4%, *p* < 0.001) (Table 1).

However, patients with GUI were significantly less likely to die within the first stay in hospital (12.0% vs. 16.2%, *p* < 0.001), which was also reflected in the RISC II score (12.8% vs. 14.1%). Mortality was not examined in relation to the different trauma center levels.

### 3.2. The Scoring System

After analyzing our patient cohort and identifying the most significant differences between patient groups with and without GUIs in a univariate analysis, we aimed to develop a novel scoring system. Therefore, we evaluated all differences which proved to be highly significant using a binary logistic regression model.

Men had higher odds of sustaining GUIs than women (OR: 1.31, 95% CI [1.22, 1.41]). Additionally, younger patients ≤ 60 years of age were significantly more likely to sustain GUIs (OR: 1.59, 95% CI [1.49, 1.71]).

As previously reported, the presence of pelvic fractures greatly increases the likelihood of GUIs. However, the severity of this pelvic injury is even more important. There was no significant difference in the odds of sustaining GUIs with moderate pelvic injuries (AIS = 2) (OR: 0.99, 95% CI [0.89, 1.10]). The odds increased steadily with rising AIS: serious pelvic injury (AIS = 3): (OR: 1.46, 95% CI [1.31, 1.64]), severe pelvic injury (AIS = 4): (OR:1.97, 95% CI [1.80, 2.16]), and critical pelvic injury (AIS = 5): (OR: 2.58, 95% CI [2.29, 2.91]).

Furthermore, injuries caused by a motorcycle accident also significantly raised the odds for sustaining GUIs (OR: 1.70, 95% CI [1.55, 1.87]).

We also found that the number of injured body regions was highly significant for sustaining GUI. The odds raised significantly with an increasing number of injured body regions: at least three regions: (OR: 1.70, 95% CI [1.55, 1.87]), four regions: (OR: 2.81, 95% CI [2.55, 3.09]), five regions: (OR: 4.20, 95% CI [3.78, 4.68]), ≥six regions: (OR: 6.63, 95% CI [5.88, 7.47]). In a second step, points were assigned for the individual risk factors based on the individual odds ratios. Applying this technique, the scoring system was designed as follows:

A maximum of 11 points can thus be achieved in the “GenitoUrinary Injuries in Polytraumatized Patients” (GUIPP)-scoring system (Table 2).

Low scores of 0–5 make the prevalence of GUIs very unlikely (<10%), while intermediate scores of 6–8 show a moderate likelihood and high scores of 9–11 have a high risk of over 28.5% for GUIs.

**Figure 3:**
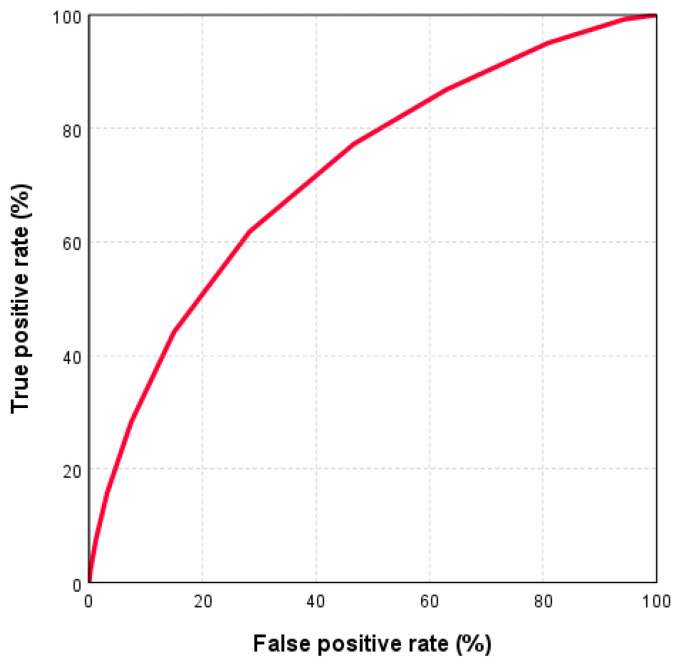
Receiver operating characteristic (ROC) curve for the use of the GUIPP scoring system in predicting genitourinary injuries in polytraumatized patients.

**Figure 4:**
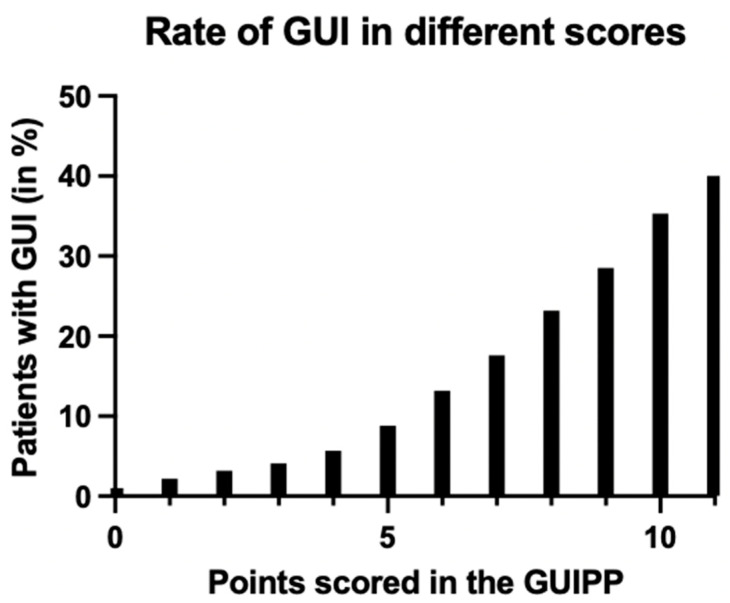
Bar graph showing the rates of genitourinary injuries (GUI) with each score achieved in the GUIPP scoring system.

Subsequently, we analyzed the validity of this test by performing a receiver operating characteristic (ROC) analysis. Here, the area under the curve (AUC) was 0.72 (95% CI: [0.71, 0.73]), indicating good discrimination (Figure 3). Figure 4 shows the percentages of GUIs at the different scores.

## 4. Discussion

To the best of our knowledge, this is by far the largest study cohort to analyze predictive factors for GUIs in severely injured patients.

As previously described, a diagnosis of GUI is frequently missed in polytraumatized patients [4]. However, the missed and delayed diagnosis of GUI in severely injured patients are associated with a lower overall outcome and higher long-term morbidity [4,14,16]. Therefore, this study aims to provide clinicians with a scoring system to predict GUIs in polytraumatized patients earlier.

With the recently introduced UPPS score, a first attempt was made towards the development of such a tool [6]. However, the UPPS scoring system was only applicable to patients with pelvic fractures and/or injuries of the thoracic or lumbar spine. This requires that these bony injuries are already known, which can make the use of the UPPS scoring system in the emergency setting difficult. Therefore, we decided to include all polytraumatized patients in developing the “genitourinary injuries in polytraumatized patients” (GUIPP) scoring system in order to simplify its handling.

Some of the independent risk factors that were highly predictive in the UPPS score also proved to be highly significant in this study population. Significantly more male patients suffered from GUIs (77.2% vs. 71.3%), which is also in accordance to the current literature [2]. This is most likely to be attributed to the different anatomy, as the female urethra is significantly shorter and, together with the female genital, less exposed than the male urethra [4]. Additionally, men show higher risk-taking tendencies, thus suffering from high-velocity trauma such as motorcycling accidents more often than women [2,17,18].

This also explains another highly predictive factor for the presence of a GUI: the trauma mechanism. Of all the registered trauma mechanisms (car/truck, bicycle, pedestrian, motorcycle, fall from ≥3 m, fall from <3 m, other), the odds for sustaining GUIs were significantly higher in patients injured in motorcycle accidents (OR: 1.70, 95% CI [1.55, 1.87]) than by any other trauma mechanism. As mentioned above, this is mainly due to the more exposed anatomy of the male reproductive organs and it is known from the literature that far more motorcyclists are men, which clearly increases the likelihood of accidents [8,17,19].

Furthermore, pelvic girdle injuries are common after high-velocity trauma such as motorcycle accidents or falls from great heights and are often associated especially with lower urogenital tract injuries [5,20]. The patients with pelvic girdle injuries were significantly more likely to sustain GUIs than patients without pelvic girdle injuries (45.0% vs. 24.2%). Additionally, the odds for sustaining GUIs rose significantly with the severity of the pelvic fracture measured by AIS. As described before, there was no correlation between GUIs and moderate pelvic girdle injuries (AIS = 2). This is easily explained by the fact that pelvic injuries with an AIS of 2 are mainly moderate, stable injuries outside of the weight-bearing areas and without major dislocations. It has been reported that the risk for sustaining bladder and urethral injuries due to pelvic fractures is mostly caused by the rupture of ligaments supporting the lower urogenital tract or, more rarely, also by piercing bony fragments [8,21]. Therefore, the wider diastasis of the symphysis such as in open-book fractures or vertical shear fractures carry a higher probability of GUIs [1,20]. This is also well represented in our study population, as patients with critical pelvic injuries (AIS 5) had the highest odds of sustaining a GUI.

The UPPS scoring system also included “gross hematuria on first presentation in the emergency room” as an independent risk factor in the scoring system. However, gross hematuria is not registered in the TR-DGU, so it was not used in constructing the GUIPP [22].

In our patient cohort, we found that patients with GUIs were significantly more severely injured than patients without GUIs, as measured by ISS (ISS 30.8 ± 13.1 vs. 26.6 ± 10.6). Eidelman and coworkers have also stated in their research that an ISS ≥ 34 was associated with an increasing number of concurrent bladder injuries after blunt trauma [13]. The ISS is calculated using the square sum of the three worst injured body regions according to the AIS scale. However, the nine body regions listed in the AIS scale do not match completely with the six body regions registered by the ISS [23]. As the AIS of the pelvic girdle injury was already considered in our scoring system as an independent factor, using ISS for the scoring system again could possibly double the influence of the pelvic AIS score. Additionally, in order to calculate the ISS properly, a correct and complete list of all diagnoses, which can often only be obtained after initial WBCT or even later on during the hospital stay, is needed. Therefore, we decided to use the number of injured body regions (with AIS ≥ 2) rather than the ISS score itself for developing our scoring system to give credit to multiple injuries. A full body check should always be conducted in the emergency room and in the field, so treating physicians will have a good idea of how many body regions are injured [24]. Furthermore, the statistical analysis showed that the number of injured body regions was much more sensitive towards predicting GUIs than an ISS score of ≥35. The odds for sustaining GUIs increased almost exponentially with a rising number of injured body regions and was therefore given more weight in the GUIPP score (Table 2). Bjurlin and coworkers have observed a similar association of patients with GUIs having a greater number of injuries in different body regions [8].

This newly introduced GUIPP scoring system has good sensitivity, with an AUC of 0.72, as high scores of ≥10 carry a 35–40% probability of the presence of GUIs. We suggest that high scores should prompt physicians to seek early urologic consultation and to extend diagnostic imaging specifically to the genitourinary system. However, we believe that the real strength of the GUIPP score lies in its very high negative predictive value, as in low scores of 0–5, the probability of a GUI is under 10%.

The development of this scoring system is also aimed at reducing missed and delayed diagnoses in order to improve the outcome of these patients. This is highly relevant as our data analysis showed that patients with GUIs had significantly higher complication rates (sepsis and MOF) and needed to be treated in hospital and in the ICU for a longer period of time. This could be due to the fact that the delayed diagnosis of GUIs could lead to early complications, such as peritonitis with intraperitoneal bladder ruptures, bleeding, or acute kidney injury [1]. Furthermore, several studies in the past have shown poorer outcome and long-term results after a delayed diagnosis of GUIs, underlining the importance of early diagnosis and management in critically injured patients [3,7,25,26].

Additionally, a trend towards the conservative treatment of GUIs has developed in recent years [1,3,27]. This, on one hand, has considerably reduced the number of surgical interventions and associated risks, but conservative treatment, for example of urethral ruptures, can take longer in the healing process [4].

While complication rates in patients with GUIs are higher, mortality is significantly lower. This could be due to the fact that the very seriously injured patients (ISS ≥ 35), which, as shown above, also tend to include patients with GUIs, were significantly more likely to be taken to level 1 trauma centers. Studies have shown that reorganizing trauma care and centralizing expertise in level 1 trauma centers has reduced mortality immensely [28]. Baloche and coworkers also demonstrated that nephrectomy rates and the failure of conservative treatment was lower in high-volume hospitals [29]. Additionally, as stated before, patients with GUIs are younger, thus, presumably healthier before trauma. However, we do not assume that older age protects from GUIs. The lower mortality in patients with GUIs was also reflected by the RISC II score in our study population.

This retrospective cohort analysis has several limitations. Due to its registry based retrospective data collection, imprecise and incomplete data sets cannot be ruled out. However, the extremely large study cohort gives smaller mistakes less weight.

Even though gross hematuria was a highly predictive factor for GUIs in the UPPS scoring system and also in the literature, it is not documented in the TR-DGU. Therefore, it could not be used in developing the GUIPP score. However, since gross hematuria is often considered the main symptom of GUIs, the awareness of treating physicians towards GUIs in the presence of gross hematuria should hopefully be extremely high anyway. The GUIPP score is designed to identify and detect the less obvious injuries of the urogenital tract and also aid physicians towards more precise indications of a delayed urographic phase required to fully assess the urogenital tract. However, a prospective validation study of the GUIPP scoring system will be needed to evaluate its clinical value.

Additionally, we believe that the identification of important risk factors in the field will contribute to an even higher proportion of patients with GUIs being transported to level 1 trauma centers, where urologists are available 24 h a day and specialized treatment can be initiated immediately.

## 5. Conclusions

As GUIs remain often-overlooked injuries in polytraumatized patients, we identified the most significant risk factors and combined these into one comprehensive scoring system: the novel “genitourinary injuries in polytraumatized patients” (GUIPP) score. It was developed using data from over 70,000 patients, and is therefore the largest study on this topic to date.

We believe that the GUIPP will make a valuable contribution to the early detection of GUIs, thereby reducing long-term morbidity and complications in severely injured patients. It is an easy-to-use tool that can be used in the emergency room as well as in the field, allowing physicians to initiate appropriate diagnosis and urological consultation early on.

## Figures and Tables

**Table 1:** Summary of categories which showed significant differences between patient populations with and without GUIs.

		With GUI (*n* = 4760)	Without GUI (*n* = 65,707)
**Patient Characteristics**			
Sex	Men	77.2%	71.3%
	Women	22.8%	28.7%
Age (years)		46.8 ± 20.1	55.1 ± 20.8
Motorcycle accident		24.7%	13.0%
ISS score		30.8 ± 13.1	26.6 ± 10.6
Number of injured body regions	≥3 regions	5.3%	94.7%
	≥4 regions	9.1%	90.9%
	≥5 regions	14.4%	85.6%
	≥6 regions	22.9%	77.1%
Severity of pelvic girdle injuries	AIS 3	11.0%	89.0%
	AIS 4	13.1%	86.9%
	AIS 5	18.7%	81.3%
**Treatment and outcome**			
Treatment on ICU		94.3%	91.0%
Length of ICU stay (days)		11.3 ± 15.3	8.4 ± 12.0
Length of stay in hospital (days)		23.9 ± 23.0	18.8 ± 19.4
MOF		32.3%	26.1%
Sepsis		11.1%	7.8%
Mortality		12.0%	16.2%

AIS: Abbreviated Injury Scale; GUI: genitourinary injury; ICU: intensive care unit; ISS: Injury Severity Score; MOF: multi organ failure.

**Table 2:** The novel “genitourinary injuries in polytraumatized patients” (GUIPP) scoring system.

		Points Awarded in the Scoring System	Odds Ratio (with 95% CI)
Male patient		1 point	1.31 [1.22, 1.41]
Age ≤ 60 years		1 point	1.59 [1.49, 1.71]
Motor cycle accident		1 point	1.70 [1.55, 1.87]
Pelvic injury	AIS 2	0 points	0.99 [0.89, 1.10]
	AIS 3	1 point	1.46 [1.31, 1.64]
	AIS 4	2 points	1.97 [1.80, 2.16]
	AIS 5	3 points	2.58 [2.29, 2.91]
Number of injured body regions	3	2 points	1.70 [1.55, 1.87]
	4	3 points	2.81 [2.55, 3.09]
	5	4 points	4.20 [3.78, 4.68]
	≥6	5 points	6.63 [5.88, 7.47]

AIS = abbreviated injury scale; CI: confidence interval.

## Data Availability

Restrictions apply to the availability of these data. Data were obtained from TraumaRegister DGU and are available from the authors with the permission of TraumaRegister DGU.

## References

[1] (2023). EAU Guidelines on Urological Trauma. EAU Guidelines Office. https://uroweb.org/wp-content/uploads/EAU-Guidelines-on-Urological-Trauma-2020.pdf.

[2] McGeady J.B., Breyer B.N. (2013). Current epidemiology of genitourinary trauma. Urol. Clin. North. Am..

[3] Coccolini F., Moore E.E., Kluger Y., Biffl W., Leppaniemi A., Matsumura Y., Kim F., Peitzman A.B., Fraga G.P., Sartelli M. (2019). Kidney and uro-trauma: WSES-AAST guidelines. World J. Emerg. Surg..

[4] Morey A.F., Broghammer J.A., Hollowell C.M.P., McKibben M.J., Souter L. (2021). Urotrauma Guideline 2020: AUA Guideline. J. Urol..

[5] Demetriades D., Karaiskakis M., Toutouzas K., Alo K., Velmahos G., Chan L. (2002). Pelvic fractures: Epidemiology and predictors of associated abdominal injuries and outcomes. J. Am. Coll. Surg..

[6] Mair O.A., Himmler M., Brunnemer S., Faymonville C., Honeck P., Horn T., Biberthaler P., Hanschen M. (2022). Positive Predictive Factors for Urogenital Injuries in Severely Injured Patients with Pelvic and Spinal Fractures: Introducing the UPPS Scoring System. Medicina.

[7] Fochtmann U., Jungbluth P., Maek M., Zimmermann W., Lefering R., Lendemans S., Hussmann B. (2022). Do concomitant urological injuries in severely injured patients lead to poorer outcomes? A multivariate risk analysis. Urologie.

[8] Bjurlin M.A., Fantus R.J., Mellett M.M., Goble S.M. (2009). Genitourinary injuries in pelvic fracture morbidity and mortality using the National Trauma Data Bank. J. Trauma.

[9] Rovere G., Smakaj A., Perna A., De Mauro D., Are L., Meccariello L., Fidanza A., Erasmo R., Falez F., Maccauro G. (2023). Correlation between traumatic pelvic ring injuries and sexual dysfunctions: A multicentric retrospective study. Int. Orthop..

[10] Persu C., Braschi E., Lavelle J. (2014). A review of prospective Clinical Trials for neurogenic bladder: The place of surgery, experimental techniques and devices. Cent. Eur. J. Urol..

[11] Karim T., Topno M., Sharma V., Picardo R., Hastir A. (2010). Bladder injuries frequently missed in polytrauma patients. Open Access J. Urol..

[12] McCombie S.P., Thyer I., Corcoran N.M., Rowling C., Dyer J., Le Roux A., Kuan M., Wallace D.M., Hayne D. (2014). The conservative management of renal trauma: A literature review and practical clinical guideline from Australia and New Zealand. BJU Int..

[13] Eidelman E., Stormont I., Churukanti G., Shreck E., Belay R., Capodice S., Maass D., Stein D.M., Siddiqui M.M. (2019). Injury severity score associated with concurrent bladder injury in patients with blunt urethral injury. World J. Urol..

[14] Pereira B.M., de Campos C.C., Calderan T.R., Reis L.O., Fraga G.P. (2013). Bladder injuries after external trauma: 20 years experience report in a population-based cross-sectional view. World J. Urol..

[15] Palmer C.S., Gabbe B.J., Cameron P.A. (2016). Defining major trauma using the 2008 Abbreviated Injury Scale. Injury.

[16] Mazzone A., Anderson R., Voelzke B.B., Vanni A.J., Elliott S.P., Breyer B.N., Erickson B.A., Buckley J., Myers J. (2021). Sexual function following pelvic fracture urethral injury and posterior urethroplasty. Transl. Androl. Urol..

[17] Eden L., Kühn A., Gilbert F., Meffert R.H., Lefering R. (2019). Increased Mortality Among Critically Injured Motorcyclists over 65 Years of Age. Dtsch. Arztebl. Int..

[18] Schoeneberg C., Kauther M.D., Hussmann B., Keitel J., Schmitz D., Lendemans S. (2013). Gender-specific differences in severely injured patients between 2002 and 2011: Data analysis with matched-pair analysis. Crit. Care.

[19] Leijdesdorff H.A., Siegerink B., Sier C.F., Reurings M.C., Schipper I.B. (2012). Injury pattern, injury severity, and mortality in 33,495 hospital-admitted victims of motorized two-wheeled vehicle crashes in The Netherlands. J. Trauma Acute Care Surg..

[20] Johnsen N.V., Dmochowski R.R., Young J.B., Guillamondegui O.D. (2017). Epidemiology of Blunt Lower Urinary Tract Trauma With and Without Pelvic Fracture. Urology.

[21] Basta A.M., Blackmore C.C., Wessells H. (2017). Predicting Urethral Injury From Pelvic Fracture Patterns in Male Patients With Blunt Trauma. J. Urol..

[22] Akademie der Unfallchirurgie GmbH (2021). Standard Sheet—V2020. https://www.traumaregister-dgu.de/fileadmin/user_upload/TR-DGU_-_Standard_form_English.pdf.

[23] Baker S.P., O’Neill B., Haddon W., Long W.B. (1974). The injury severity score: A method for describing patients with multiple injuries and evaluating emergency care. J. Trauma.

[24] The ATLS Subcommittee, American College of Surgeons’ Committee on Trauma, The International ATLS Working Group (2013). Advanced trauma life support (ATLS^®^): The ninth edition. J. Trauma Acute Care Surg..

[25] Kunkle D.A., Kansas B.T., Pathak A., Goldberg A.J., Mydlo J.H. (2006). Delayed diagnosis of traumatic ureteral injuries. J. Urol..

[26] Lee S.H., Bak C.W., Choi M.H., Lee H.S., Lee M.S., Yoon S.J. (2008). Trauma to male genital organs: A 10-year review of 156 patients, including 118 treated by surgery. BJU Int..

[27] Sujenthiran A., Elshout P.J., Veskimae E., MacLennan S., Yuan Y., Serafetinidis E., Sharma D.M., Kitrey N.D., Djakovic N., Lumen N. (2019). Is Nonoperative Management the Best First-line Option for High-grade Renal trauma? A Systematic Review. Eur. Urol. Focus.

[28] MacKenzie E.J., Rivara F.P., Jurkovich G.J., Nathens A.B., Frey K.P., Egleston B.L., Salkever D.S., Scharfstein D.O. (2006). A national evaluation of the effect of trauma-center care on mortality. N. Engl. J. Med..

[29] Baloche P., Szabla N., Freton L., Hutin M., Ruggiero M., Dominique I., Millet C., Bergerat S., Panayotopoulos P., Betari R. (2022). Impact of Hospital Volume on the Outcomes of Renal Trauma Management. Eur. Urol. Open Sci..

